# Co-Occurring Subacute Combined Degeneration and Guillain-Barré Syndrome Resulting From Nitrous Oxide Abuse: A Case Report

**DOI:** 10.7759/cureus.32756

**Published:** 2022-12-20

**Authors:** Kristen Strom, Ateaya Lima, Vahideh Tabarzadi

**Affiliations:** 1 Psychiatry, Ross University School of Medicine, Bridgetown, BRB; 2 Psychiatry, Jamaica Hospital Medical Center, New York City, USA; 3 Psychiatry, Yale University, New Haven, USA

**Keywords:** case report, whippets, anxiety disorder, adjustment disorder, guillain-barre syndrome, subacute combined degeneration, addiction, nitrous oxide

## Abstract

Growing in prevalence as a recreational drug of abuse across a broad scope of people, nitrous oxide (N_2_O) has proven to be a public health concern. Side effects of its abuse have a great degree of variation among people ranging from neurologic, psychiatric, and medical symptoms. We present a rare case of a 23-year-old female with a history of N_2_O abuse, who presented with symptoms of both subacute combined degeneration syndrome (SCD) and Guillain-Barré syndrome (GBS). She presented with three weeks history of progressive bilateral lower extremity weakness, burning and tingling sensation, difficulty walking, and falls. This patient underwent an extensive medical workup upon hospitalization. Notable findings of the magnetic resonance imaging (MRI) of her cervical spine showed an abnormal cervical cord signal intensity pattern typical for vitamin B_12 _deficiency and her lumbar puncture showed albuminocytological dissociation, which confirmed the diagnosis of GBS. For these conditions, this patient was successfully treated with weekly vitamin B_12_ injections and five treatments of plasmapheresis. Her condition was additionally complicated by worsening anxiety and depression, which were treated with gabapentin, escitalopram, trazodone, hydroxyzine, and melatonin. She demonstrated great clinical improvement with almost complete resolution of her symptoms at the time of her hospital discharge. This case highlights the easy accessibility, addiction potential, and adverse consequences related to N_2_O abuse.

## Introduction

Nitrous oxide (N_2_O) is a colorless, odorless gas at room temperature and atmospheric pressure. It produces a state of analgesia, depersonalization, derealization, dizziness, euphoria, and sound distortion [1-5]. This drug was first discovered for its euphoric effects in 1772 and was first used medicinally in 1844 for its anesthetic properties [1,3,6]. In recent years, N_2_O has grown in popularity as a recreational drug of abuse [4]. It is believed that the sought effect of euphoria is one of the greatest factors contributing to its abuse [7,8].

N_2_O is commonly inhaled from a steel bulb known as a “whippet” with the use of a commercial-grade whipped cream dispenser colloquially referred to as the “cracker” [1,2,7-9]. A standard whippet contains 8g of 100% N_2_O and can be purchased in bulk on the internet at a very affordable price [1-4,7]. This is designed to be used in the food industry for the use of commercial-grade whipped cream due to its bacteriostatic and propellant properties [1,2,5,7,10].

N_2_O-filled balloons and whippets are becoming more mainstream and increasingly available in social settings such as festivals, clubs, raves, and house parties [2,3,8]. Most users dispense the gas into a balloon and inhale from the balloon [2,4]. N_2_O can also be extracted from prepackaged aerosolized whipped cream canisters, each containing 3 liters of >87% N_2_O gas, and are commonly sold in grocery and convenience stores at an affordable price [4,7,10-13]. 

N_2_O's association with the development of vitamin B_12_ deficiency is well documented in the literature with case reports becoming increasingly common [1-4,6,8,11,13]. Some reports have shown that vitamin B_12_ deficiency can potentially “mimic” rare neurologic conditions [3]. We present a rare case of subacute combined degeneration syndrome (SCD) and Guillain-Barré syndrome (GBS), co-occurring in a 23- year-old female with a history of N_2_O abuse, additionally complicated by worsening anxiety and depression.

This case report was previously presented as an e-poster at the 2021 European Association of Psychosomatic Medicine annual conference on June 2-5, 2021. 

## Case presentation

A 23-year-old female, previously healthy with a past psychiatric history of attention deficit hyperactivity disorder (ADHD), depression, and polysubstance abuse, including recent increased N_2_O use, presented to the hospital with a three-week history of bilateral lower extremity weakness, burning sensation, paresthesia, difficulty walking, and falls.

She had an extensive medical workup consisting of labs, imaging studies, and evaluations for infectious diseases, inflammatory causes, and autoimmune syndromes (Table 1). Her neurological examination showed decreased motor strength in bilateral upper extremities, bilateral foot drop, and decreased sensation and reflexes of bilateral upper and lower extremities. Her pertinent positive imaging and laboratory findings consisted of MRI of the cervical spine showing an abnormal cervical cord signal intensity pattern as well as a lumbar puncture showing albuminocytological dissociation (Table 1).

**Table 1:** Summary of medical workup MRI: magnetic resonance imaging; CSF: cerebral spinal fluid; EBV: Ebstein-Barr virus; VZV: varicella zoster virus; CMV: cytomegalovirus; HIV: human immunodeficiency virus; Mg: magnesium; Phos: phosphate; ANCA: antineutrophil cytoplasmic antibodies; anti-dsDNA: anti-double stranded deoxyribonucleic acid; SSA: SS-A/Ro Ab, IgG, S; SSB: SS-B/La Ab, IgG, S; ACE: angiotensin-converting enzyme; RF: rheumatoid factor; TPO ab: thyroid peroxidase antibody; C3: complement C3; C4: complement C4; IgA: immunoglobulin A; SPEP: serum protein electrophoresis; MMA: methylmalonic acid

Pertinent Positives	Pertinent Negatives
MRI consistent with SCD changes from Vitamin B_12_ deficiency in dorsal columns on cervical-spine imaging	Mycoplasma, EBV, VZV, CMV, enteroviruses, West Nile, influenza, dengue, zika, Lyme, HIV, hepatitis panel
CSF Albuminocytologic dissociation	Mg, Phos, ANCA, B1, B6, ANA, anti-dsDNA, SSA, SSB, ACE, RF, TPO ab, thyroglobulin ab, Ceruloplasmin levels, C3, C4, Cryoglobulinemia, IgA, SPEP, Antiphospholipid panel, D-dimer, Sickle cell screen, MMA, Parietal cell ab, Celiac disease panel, Gastrin, Vitamin E
Vitamin B_12_ level = 96 LOW	Arsenic, heavy metal screening

With these findings, she was diagnosed with SCD and GBS secondary to N_2_O abuse. Regarding SCD, she was successfully treated with weekly B_12_ injections. For GBS, she received five treatments of plasmapheresis. She was also started on gabapentin 300mg, oral, three times a day, for the dual function of helping with neuropathy-associated symptoms and for its anxiolytic properties. Medically, her symptoms improved; however, she continued to demonstrate lower extremity weakness warranting transfer to inpatient physical rehabilitation.

During her stay in inpatient physical rehabilitation, the patient endorsed worsening depression, insomnia, intermittent periods of anxiety, palpitations, and increased irritability. The consultation-liaison (C-L) psychiatry team was called to evaluate these symptoms. Upon evaluation, she denied any mania, perceptual disturbances, or suicidal/homicidal ideation, intent, or plan. She was diagnosed with adjustment disorder with depressed mood and was managed with psychotherapy, in addition to escitalopram 20mg oral daily, trazodone 25mg oral at bedtime, hydroxyzine 25mg oral every eight hours as needed for anxiety, melatonin 3mg oral at bedtime, and gabapentin 300mg oral three times a day. Her anxiety and depression improved with this regimen. She was discharged home on weekly intramuscular Vitamin B_12_ injections, in addition to her psychiatric medications. She was also provided with a referral to outpatient rehabilitation for continued physical therapy and a referral for outpatient psychiatric services. However, she declined follow-up with substance use rehabilitation.

Psychiatric history

The patient was an unemployed, single, childless female who rented an apartment with friends. She had a history of ADHD that was diagnosed at age 13, with trials of multiple stimulants and subsequent failure due to side effects of excessive sedation and decreased appetite. She had a remote history of on-again, off-again depression since her teenage years, often exacerbated by a strained relationship with her mother. She was inconsistently adherent to outpatient therapy and was never prescribed any antidepressants. Of note, the patient had a family history of a mother with alcoholism.

The patient was first introduced to various substances at the age of 18. She spent her time socializing with her housemates and friends, often in the form of recreational drug use, frequenting the rave and party scene. She admitted to using alcohol recreationally, cigarettes, e-cigarettes, marijuana, cocaine, ecstasy, and whippets with increasing use while quarantined during the coronavirus disease 2019 (COVID-19) pandemic. She admitted to increasing the use of whippets just prior to the onset of her symptoms; however, she was unable to quantify the amount or frequency of use, only stating that it was “continuous.” The patient had poor insight into her polysubstance use, especially her abuse of N_2_O. She referred to it as “something (she) does with friends.”

## Discussion

N_2_O exerts its euphoric, analgesic, and addictive effects through its actions on the opioid receptors mu, kappa, and delta, in addition to dopamine D2-like receptors, and GABAergic neurons [8,10,11]. The euphoric and anxiolytic effects are rapid in onset, reportedly taking approximately 10 seconds following inhalation, and typically resolve within only a few minutes after inhalation [8]. For sustained euphoria, continuous drug use is required. Most moderate users report using less than 10 balloons per session, resulting in minimal side effects [8]. However, for the population of heavy users, with the use of more than 50-100 bulbs per session or prolonged use, the side effects are much more serious and persistent [2,8]. This subpopulation of heavy users is also more likely to develop a dependence on N_2_O in a dose-response manner [2].

Vitamin B_12_ deficiency in N_2_O users often presents as SCD with rapid onset [9,14,15]. By definition, this is the degeneration of the posterior and lateral spinal columns [16]. Common presenting complaints include numbness, paresthesia, and/or weakness of the lower extremities [1,7]. Vitamin B_12_ deficiency secondary to N_2_O abuse has been shown to develop within two days to weeks of the onset of use. This rapid development is due to N_2_O’s mechanism of oxidation. This is in contrast to vitamin B_12_ deficiency of other causes, such as inadequate intake, which may take years to develop [3].

The mechanism by which N_2_O causes SCD is through the alteration of a series of biochemical pathways in which vitamin B_12_ is a cofactor. The N_2_O molecule oxides vitamin B_12_ cobalt ion from the 1^+^ to 3^+^ valence state; this inactivates methylcobalamin. This leads to the dysfunction of methionine synthase not being able to convert homocysteine to methionine, leading to decreased myelin production and resulting in increased homocysteine levels. DNA synthesis is also affected by the inability to convert 5-methyltetrahydrofolate to tetrahydrofolate. Lastly, methyl-malonyl Co-A mutase cannot effectively convert methyl-malonyl Co-A to succinyl Co-A resulting in increased levels of methylmalonic acid (Table 2) [1,5,6,9,13].

**Table 2:** Comparison between subacute combined degeneration and Guillain-Barré syndrome LE: lower extremity; UE: upper extremity; CBC: complete blood count; HCY: homocysteine; MMA: methylmalonic acid Megaloblastic Madness: neuropsychiatric manifestations, such as altered personality, memory loss, depression, hypomania, paranoid psychosis with auditory and visual hallucinations, reported with vitamin B_12_ deficiency References: Sethi et al. [9], Means et al. [16], Chandrashekhar and Dimachkie [17]

	Subacute Combined Degeneration	Guillain-Barre Syndrome
Mechanism	Oxidation of Vitamin B_12_ 1^+^ --> 3^+^, Depletes available Vitamin B_12_ for biochemical pathways – decreases myelin synthesis	Overactive immune system damaging the peripheral nervous symptoms, molecular mimicry
Neurologic Presentation	Megaloblastic anemia, loss of vibratory sensation, tactile sensation, position discrimination (LE>UE), increased tone (spasticity), memory loss, drowsiness, confusion, lower extremity weakness, paresthesia, ataxia	Rapid ascending flaccid paralysis starting in lower extremity, absent/depressed deep tendon reflexes, lower extremity weakness, paresthesia, ataxia
Psychiatric Presentation	“Megaloblastic Madness”	
Work Up	CBC and peripheral blood smear, vitamin B12 levels, HCY, MMA, macrocytic anemia, hypersegmented neutrophils	Lumbar puncture: albuminocytologic dissociation, electrodiagnostic studies: demyelination

In the United States, because N_2_O is not classified as a controlled substance, the Drug Enforcement Administration (DEA) does not regulate it. This further aids in the easy accessibility to users seeking to use N_2_O as a drug of abuse. Individual states have attempted to pass legislation that regulates the commercial possession, sale, and distribution of N_2_O with the goal of decreasing misuse. However, surveys from the Centers for Disease Control and Prevention (CDC) have demonstrated the opposite with increasing rates of misuse [4].

A systematic literature review performed by Garakani et al. in 2016 highlights manifestations of N_2_O abuse [1]. This review found 91 cases of N_2_O abuse-related complications; 72 of these cases presented with primary neurological symptoms consisting of “weakness,” “numbness,” and “paresthesia." Primary psychiatric complaints were documented in 11 of the cases with delusions being the most prevalent symptom. The final eight cases presented with other medical sequelae including pneumomediastinum, pulmonary toxicity, and frostbite. There were also 11 additional case reports in which N_2_O was found to be the primary cause of death by means of hypoxia and subsequent asphyxia. Of the 91 cases reported in this review, most symptomatic cases were associated with low vitamin B_12_ levels [1].

While some literature suggests full reversibility of neurologic and psychiatric symptoms, several reports have demonstrated that even with vitamin B_12_ being repleted intramuscularly, the symptoms are not fully reversible, and patients have residual lifelong symptoms [3,5,14].

Our patient was additionally diagnosed with GBS, which is an inflammatory condition resulting from an overactive immune system damaging the peripheral nervous system [17]. No clear mechanism has been proposed to explain the development of GBS in the setting of N_2_O abuse. One possible explanation for the development of GBS may be that N_2_O is neurotoxic in its antagonistic effects at N-methyl-D-aspartate (NMDA) receptors [1]. This leads to imbalances in homocysteine resulting in oxidative stress that leads to demyelination [1]. Common presenting symptoms of GBS are weakness, ascending paralysis, and decreased deep tendon reflexes (Table 2) [17]. These symptoms are a direct result of the demyelination of the peripheral nerves seen with this condition [17]. 

## Conclusions

N_2_O is being abused across multiple populations, representing a significant public health concern in the world today. This abuse is due to its easy accessibility and addiction potential and has very serious consequences, which as demonstrated by our case, include SCD, GBS, and exacerbation of psychiatric symptoms. While predictive factors for the development of complications may include N_2_O abuse intensity and chronicity, there is no clear set of risk factors to date that can be used to predict the type or severity of complications that may develop. There is also no formal screening tool for the detection of N_2_O abuse, which leads to missing or delaying diagnosis. It is our goal that, with the shared knowledge of this case report, the medical community is more equipped to promptly recognize and diagnose N_2_O abuse and related conditions such as SCD and GBS.
